# Regorafenib combined with sintilimab as second-line treatment for advanced HCC patient: a case report

**DOI:** 10.3389/fonc.2023.1256137

**Published:** 2023-10-10

**Authors:** Yanzhi Wan, Hong Zhu

**Affiliations:** Department of Medical Oncology, Cancer Center, West China Hospital, Sichuan University, Chengdu, Sichuan, China

**Keywords:** hepatocellular carcinoma, lenvatinib, sintilimab, regorafenib, second-line treatment

## Abstract

Hepatocellular carcinoma (HCC) is a tumor with a high degree of malignancy. Patients have poor outcomes and short survival times, especially after progression to first-line systemic therapy. Regorafenib is the standard second-line treatment for HCC, but there is no conclusive evidence that regorafenib combined with immunotherapy can be used as a second-line treatment. We present the case of a 50-year-old man with a chronic hepatitis B (CHB) infection for more than 30 years who was diagnosed with stage Barcelona Clinic Liver Cancer (BCLC)-B hepatocellular carcinoma. Unfortunately, recurrence and metastasis occurred soon after radical surgery and standard first-line treatment with lenvatinib. The patient was then treated with regorafenib plus sintilimab as second-line treatment. Surprisingly, soon after treatment, the patient reached a state of partial response (PR) that lasted for more than one year, which is currently close to that of complete response (CR). Regorafenib combined with sintilimab as second-line treatment showed an excellent curative effect in this patient, who had HCC with multiple metastases to the liver and lungs. This treatment, which has tolerable side effects, enabled the patient to reach a state of PR that lasted over one year, which is currently close to CR. Therefore, it may be a potential second-line treatment strategy.

## Introduction

Hepatocellular carcinoma (HCC) accounts for 75%-85% of primary liver cancers (PLC) which is a common cancer worldwide with bad prognosis and maybe associated serious complications such as tumor rupture, and so on. It is the fourth leading cause of cancer-related deaths worldwide, and generally occurs in the context of chronic liver disease at the cirrhosis stage (1, 2). Patients with HCC are usually diagnosed in advanced stages, with very limited therapeutic options and bleak outcomes. Until 2007, sorafenib (a multi-target tyrosine kinase inhibitor) was first approved by the Food and Drug Administration (FDA) for the first-line treatment of advanced HCC (3).

The treatment of HCC has progressed rapidly since 2017, and an increasing number of protocols have been applied in clinical practice. Combination therapy has emerged as a promising treatment for HCC and has improved survival outcomes. Atezolizumab + bevacizumab, camrelizumab + apatinib, sintilimab + bevacizumab, and durvalumab + tremelimumab have been approved as first-line standard treatments (4, 5). Systemic therapeutic strategies for HCC currently focus on combinations of different types of targeted drugs with immunotherapies as first- or second-line treatments. In addition, systemic and locoregional treatments [transarterial chemoembolization (TACE), hepatic artery infusion chemotherapy (HAIC), ablation, and radiotherapy] greatly improve patient prognosis (6).

Regorafenib alone is the standard second-line treatment for HCC, however, there is no strong evidence that regorafenib combined with immunotherapy is effective as a second-line treatment for HCC. Here, we report a rare case of HCC that relapsed soon after surgery and first-line lenvatinib treatment. However, the patient showed a nearly complete response after more than one year of treatment with sintilimab plus regorafenib.

## Case presentation

**Figure 1:**
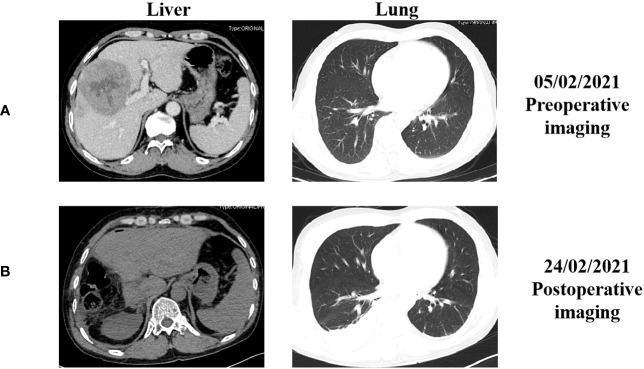
The representative abdominal and chest enhanced computed tomography (CT) images before and after the surgery. **(A)** CT images before surgery performed on February 5, 2021; **(B)** CT images after surgery performed on February 24, 2021.

**Figure 2:**
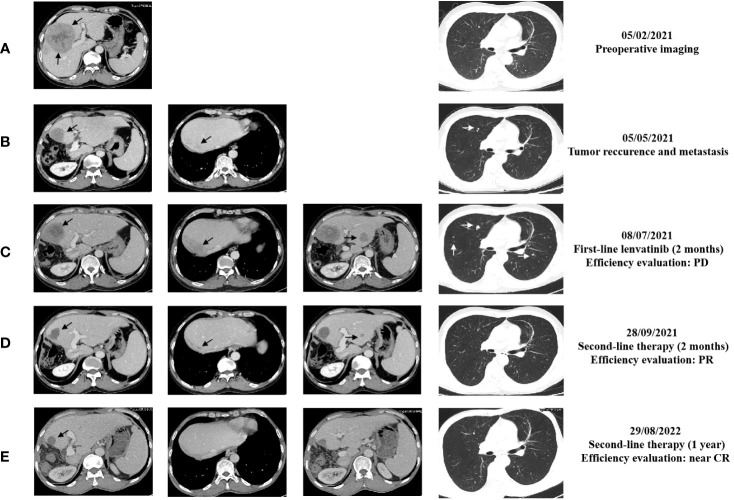
The representative abdominal and chest enhanced CT images performed at different time of the treatments. **(A)** CT images before surgery performed on February 5, 2021; **(B)** CT images performed on May 5, 2021; **(C)** CT images performed on July 8, 2021; **(D)** CT images performed on September 28, 2021; **(E)** CT images performed on August 29, 2022. The black arrows point to the liver lesions. The white arrows point to lung lesions.

A 50-year-old man with chronic hepatitis B (CHB) infection for over 30 years was diagnosed with Barcelona Clinic Liver Cancer (BCLC) stage B HCC in February, 2021. Abdominal contrast-enhanced computed tomography (CT) revealed a slightly low-density mass with a size of 8.6 cm x 8.4 cm in the junction area of the left and right lobes of the liver. Contrast-enhanced chest CT revealed no signs of metastasis before surgery (**Figures 1A**, **2A**). On February 20, 2021, the patient received a radical right complex liver cancer resection and cholecystectomy. Post-surgical pathology indicated two tumor masses in the liver (8.9 cm and 3.0 cm in diameter). The lymph nodes were negative. The histology type was medium-to-low-differentiation HCC. The tumor margins were negative. There was no major vascular invasion, and microvascular invasion (MVI) was positive, with a number greater than five. There was no invasion of the peripheral nerves. The results of immunohistochemistry staining were: GPC-3 (+), GS(+), ARG (+), AFP (+), CD34 (+), confirming the diagnosis of HCC. The CT examination on the fourth day after surgery revealed a partial absence of the liver, a slight roughness of the residual liver margin, a slightly reduced density of the adjacent liver parenchyma, and a small pleural effusion on both sides (**Figure 1B**).

Unfortunately, enhanced CT performed in May 2021 confirmed a new mass with enhancement, sized 3.9 cm × 2.7 cm, at the edge of the residual liver with the appearance of scattered, multiple, sized 0.3 cm - 0.5 cm lesions in both lungs (**Figure 2B**). In addition, tumor markers such as serum alpha-fetoprotein (AFP) and protein induced by vitamin K absence/antagonist-II (PIVKA-II) were far beyond the normal range. Hence, on May 13, 2021, the patient began to receive standard first-line treatment with oral lenvatinib, 12 mg daily. Nevertheless, in July 2021, the CT scan performed two months after the beginning of therapy showed multiple slight contrast enhancement masses and nodules in the residual liver, with a maximum of approximately 6.5 cm x 5.2 cm, and both the number and volume of lung lesions increased (**Figures 2C**). In addition, tumor marker levels continued to increase rapidly. The efficacy evaluation was progression disease (PD).

Considering the poor differentiation of malignancy and rapid progression of the disease, the patient was treated with sintilimab immunotherapy plus regorafenib-targeted therapy as second-line treatment. From July 2021 to the present, the patient has regularly received the following treatment: Sintilimab 200 mg, intravenously every three weeks (total two years) and Regorafenib 120mg, take it every three weeks and stop for one week. After two months, a routine CT examination revealed that the lesions significantly reduced in the liver; the largest one was about 3.3 cm x 3.1 cm; meanwhile, the lung lesions decreased in size, to a diameter of approximately 0.2 - 0.5 cm (**Figures 2D**) compared with images in July, 2021. Furthermore, there was a decrease in tumor markers; the qualitative value of PIVKA-II decreased from 64315 mAU/ml to 22 mAU/ml, and the qualitative value of AFP decreased from 50500 ng/ml to 57.1 ng/ml. The treatment efficacy evaluation was partial response (PR).

During treatment, the treatment efficacy was evaluated as continued PR every time. In August 2022, the enhanced CT showed that there was only a slightly low-density shadow about 2.1 cm in diameter left in residue liver, without enhancement, the metastasis of both lungs basically disappeared (**Figure 2E**), and the tumor markers approached the normal value. The treatment efficacy evaluation was close to complete response (CR). We suggested the patient to take a PET-CT examination and then take surgery if there were residual tumors. However, the patient refused the PET-CT examination or repeated surgery because he couldn’t afford it. During treatment with regorafenib plus sintilimab, patients experience adverse reactions such as gum bleeding, diarrhea, and even bloody stools. After symptomatic treatment, adverse reactions were controlled. Until now, the patient continues to use and benefit from this treatment regimen.

**Figure 3:**
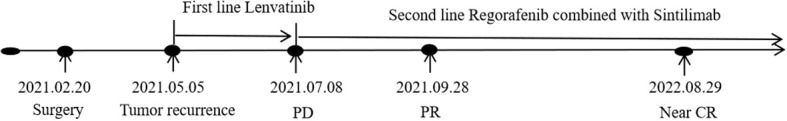
The schematic treatment timeline of the patient. PD, progression disease; PR, partial remission; CR, complete remission.

Currently, the patient has survived for more than two year after the rapid progression of first-line treatment and has achieved a good therapeutic effect without serious treatment-related adverse reactions during second-line treatment. The schematic treatment timeline is depicted in **Figure 3**.

## Discussion

Hepatocellular carcinoma (HCC) is one of the main causes of cancer-related deaths worldwide, with limited treatment options and few effective drugs available. Treatment selection mainly depends on the tumor stage (7). The BCLC staging system is most commonly used to select the best treatment strategy worldwide. Approximately half of the patients with HCC are diagnosed at an advanced stage without a chance of curative treatment and have to receive systemic therapies. In the past few decades, only a few chemotherapy and targeted therapy drugs have proven to be effective for advanced disease. The choice of treatment options was limited. In recent years, breakthroughs have been achieved in the treatment of HCC.

Hepatic resection, radiofrequency ablation, and liver transplantation are preferred for patients with early and intermediate HCC (8). Patients who undergo resection have the best chance of long-term survival, with a 5-year survival rate of 70% to 80% and a 5-year recurrence rate of 50% to 70% (9). The first peak of recurrence occurs approximately one year after resection, and a high level of AFP is predictive of a poor prognosis (10). The patient was diagnosed with BCLC-B disease with a giant liver lesion. Preoperative neoadjuvant therapy might have had a better therapeutic effect, but the patient chose to undergo direct surgical treatment. However, only two months after resection, enhanced CT confirmed intrahepatic recurrence and pulmonary metastases.

Sorafenib, an oral multi-kinase inhibitor, was the first agent to offer a survival benefit in advanced HCC and was approved by the FDA as a first-line treatment. Until now, many first-line standard treatments for HCC have been approved (11). Different patients may have individualized treatment plans. Lenvatinib, another oral multikinase inhibitor, is noninferior to sorafenib: median overall survival (OS) was 13.7 months with lenvatinib *vs*. 12.3 months with sorafenib. In the phase III REFLECT trial, lenvatinib showed remarkable effects, increasing the objective response rate to 24.1% and extending the time to progression to 8.9 months (12). In addition, lenvatinib significantly prolonged median OS to 15.0 months and median progression-free-survival to 8.4 months in the Chinese population, according to a native clinical trial (13). Hence, we chose lenvatinib as the first-line treatment. However, during the 2-month lenvatinib treatment period, the patient’s condition worsened. Enhanced CT revealed recurrent lesions in the liver, and metastatic lesions in the lungs had increased in quantity and volume. The PIVKA-II and AFP levels increased rapidly.

Drugs such as regorafenib, ramucirumab, and cabozantinib have been approved as second-line treatments for advanced HCC with strong evidence. Since the emergence of immune checkpoint inhibitors, an increasing number of clinicians have focused on the combination of immunotherapy and targeted therapy, which appears to boost the response (14). Due to rapid progression, we added sintilimab to regorafenib as a second-line treatment for this patient. Sintilimab, a native PD-1 inhibitor, was developed by Innovent Biologics and Eli Lilly. Regorafenib, a multi-kinase inhibitor, is structurally similar to sorafenib. This strategy is not a standard second-line therapy. Surprisingly, after only two months of treatment, the recurrent lesions in the liver and the metastatic lesions in the lungs were well controlled. In addition, the levels of tumor markers decreased rapidly, approaching normal values. In August 2022, the re-examination results showed that the liver recurrence continued to shrink, and the metastasis to both lungs disappeared. The patient is currently receiving regular treatment. The evaluation of comprehensive cancer control efforts has shown a continuous PR for more than one year and close to CR.

In the present case, two challenging events occurred during treatment. Recurrence and metastasis occurred quickly after surgery, and the condition progressed rapidly despite first-line standard treatment. Few reports have focused on the successful second-line treatment of advanced HCC with regorafenib plus sintilimab. The patient achieved PR for more than one year, which was close to CR. Besides, the side effects were mild and tolerable.

## Conclusions

With the development of immunotherapy, breakthroughs have been made in HCC treatment. However, HCC has a poor prognosis. In this case, the disease progressed rapidly after surgery and first-line lenvatinib treatment which showed high malignancy. After the use of regorafenib combined with sintilimab as second-line treatment, the patient quickly achieved PR. To date, the continuous PR status of the patient has lasted for more than one year, which is close to CR. The combination of regorafenib and sintilimab may be a better second-line treatment for HCC. However, further prospective studies are required to confirm this hypothesis.

## Data availability statement

The original contributions presented in the study are included in the article/supplementary materials. Further inquiries can be directed to the corresponding author.

## Ethics statement

The report was approved by the West China Hospital institutional review board, and the patient provided written informed consent. The studies were conducted in accordance with the local legislation and institutional requirements. The participants provided their written informed consent to participate in this study. Written informed consent was obtained from the individual(s) for the publication of any potentially identifiable images or data included in this article.

## Author contributions

HZ: Writing – review & editing. YW: Writing – original draft.
